# Permeability regain and aqueous phase migration during hydraulic fracturing shut-ins

**DOI:** 10.1038/s41598-018-38211-0

**Published:** 2019-02-12

**Authors:** Shuai Li, Qun Lei, Xin Wang, Bo Cai, Guangfeng Liu, Long Wang

**Affiliations:** 1 Research Institute of Petroleum Exploration & Development, PetroChina, Beijing, 100083 China; 2 Education Ministry Key Laboratory of Petroleum Engineering, China University of Petroleum, Beijing, 102249 China; 3 The 4th oil production plant in North China Oilfield, PetroChina, Langfang, 065007 China

## Abstract

Hydraulic fracturing has become a key technology to economically extract oil and gas from unconventional reservoirs. During hydraulic fracturing, fluid loss and water invasion into formation can cause serious permeability reduction near fracture face. At the same time, field practice also showed that well shut-ins after hydraulic fracturing could significantly increase hydrocarbon outputs, whereas the inner mechanism still remains unknown. In this paper, firstly, we studied permeability reduction after water invasion and permeability enhancement after well shut-ins using a core flooding system. Then, to investigate the inner mechanism, we studied aqueous phase migration during shut-ins using nuclear magnetic resonance (NMR) method. Results showed that fluids invasion reduce matrix permeability while well shut-ins can improve permeability and this improvement depends on the length of shut-ins time. NMR results showed that aqueous phases mainly distribute in macropores and mesopores after water invasion, while in shut-ins period, these invaded aqueous phases redistribute and migrate from larger pore spaces to smaller ones. Aqueous phase redistribution and migration during shut-ins period can remove near fracture water-block, reduce capillary discontinuity and increase the relative permeability of hydrocarbon phase, and this is the reason for permeability enhancement and hydrocarbon output increase after well shut-ins.

## Introduction

In recent years, the shale gas revolution in North America has made the development of unconventional oil and gas a research a hot spot, this great achievement is mainly due to the key technology breakthrough such as horizontal well drilling and multistage hydraulic fracturing^1,2^. To economically extract oil and gas from unconventional formations, a large amount of fracturing fluids is pumped into underground to create complex fractures and maximize the contact area with formation^3,4^. While it was reported that only 5–20% of fracturing fluids can be recovered during the flow-back process and this phenomenon has caught scholars’ attention^5,6^.

Explanations have been put forward such as injected fluids would be trapped near fracture face due to high capillary pressure of the formation matrix or directly trapped within fracture itself ^7–9^. Some other scholars hold the idea that imbibition or dialysis dominated by capillary pressure or osmotic pressure is another important influencing factor^10–12^. Fluid loss and water invasion into formation can cause serious permeability damage via clay swelling, solid precipitation and fines migration, this will block the original pore spaces and result in physical permeability reduction^13–15^. What’s more, capillary trapping, near fracture water-block and relative permeability reduction can also be the potential killer for oil and gas production^16–18^. At the same time, scholars also find that hydrocarbon production in Marcellus has significantly improved after undergo 6 months’ shut-ins after hydraulic fracturing^19^. Scholars always explain this phenomenon by near fracture water-block removal or aqueous phase redistribution due to capillary dominated spontaneous imbibition. During well shut-ins, aqueous phase enters porous media and displace hydrocarbon phase via counter-current imbibition, decreasing water saturation and increasing the relative permeability of hydrocarbon phase^20–23^. When near fracture water-block was removed via aqueous phase redistribution, oil and gas can seepage easily into hydraulic fracture and wellbore. However, these explanations are mainly hypothesis or based on previous experience, no convincing pore scale or visible evidence has been published to demonstrate the underlying mechanism.

Traditional methods to describe porous media structure include mercury injection method, scanning electron microscopy (SEM) method, low-pressure nitrogen adsorption-desorption method^24,25^, pressure pulse/step decay measurements^26^, and NMR method^27^, etc. Low magnetic field NMR method has widely been used in the detection of pore water content and pore fluids distribution in unconventional core samples. Scholars also tried to obtain the relationship of pore radius distribution from transverse relaxation time (T_2_) and from conventional mercury injection method considering these two methods can describe pore structures^28,29^. Meanwhile, assuming pore spaces can be simplified to be columnar or spherical, numerous studies gave a linear relation between T_2_ and pore radius^30–32^. Based on these technologies, scholars begin to use this technique to monitor water profile during imbibition in tight sandstone, shale or other unconventional rock samples^33^.

In this paper, firstly, we investigated permeability reduction after fluids invasion and permeability enhancement after well shut-ins using a core-flooding system. Then we studied aqueous phase migration during well shut-ins using NMR method. By combining these two experimental methods, we not only evaluate permeability evolution during shut-ins but also try to explain the underlying mechanism why well shut-ins can enhance permeability and hydrocarbon production.

## Methodology

Fluid loss and aqueous phase invasion into formation during hydraulic fracturing can cause serious damage to matrix permeability and hydrocarbon production, whereas well shut-ins increase it. In this paper, we built a physical model to simulate the whole process of hydraulic fracturing including water invasion, well shut-ins and flow-back by injecting different fluids from different ends of rock samples. By detecting pressure across core sample and the fluids flow rate, we calculate relative permeability reduction after water invasion and permeability regain after well shut-ins. What’s more, to explain the inner mechanism for permeability enhancement after well shut-ins, we use NMR method to detect aqueous phase migration during well shut-ins.

### Rock samples

Unconventional rock samples used in our experiment were obtained from Chang7 formation, Changqing Oil Field, China. Six cylindrical water-wet rock samples were used in our work, the first four samples were used in permeability evaluation experiment and the latter two samples were used in NMR tests. Firstly, rock sample porosity was measured using a helium sycometer, rock sample permeability was then measured using pulse decay permeability apparatus via nitrogen injection method. According to Chakraborty’s modified pulse-decay measurement approach for tight/low permeability samples, the pore pressure and confining pressure adopted in our experiment is 8 MPa and 10 MPa respectively^34^. Detailed petrophysical characteristics and the contrastive experimental scheme are given in Table 1.

**Table 1:** Physical properties and experimental scheme for the rock samples.

Rock sample	Length/cm	Diameter/cm	Porosity/%	Permeability/mD	Shut-ins time/h	Inject volume	Purpose	Lithology
cq001-1	5.01	2.51	10.2	0.21	6	1PV	Permeability evolution	Sandstone
cq001-2	5.06	2.53	10.6	0.25	12			
cq001-3	5.03	2.49	11.2	0.27	18			
cq001-4	4.96	2.52	9.7	0.19	24			
cq002-1	5.51	2.51	9.3	0.11	Periodically	0.2 PV	NMR test	Sandstone
cq002-2	5.50	2.48	9.6	0.16				

**Figure 1:**
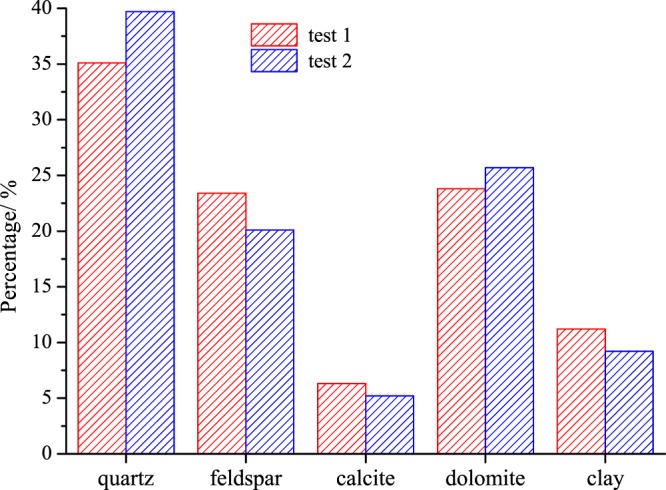
Mineral composition of rock samples. (1) Quartz is the main component of non-clay minerals. (2) Clay content is between 9–11%, not that particularly high.

In addition, some crushed rocks drilled from the same formation were used to determine their mineralogical composition using an X-ray diffraction (XRD) method and the results were shown in Fig. 1. It has shown that quartz, feldspar, calcite, and dolomite are the main components of non-clay minerals in which quartz takes the largest proportion. It should also be noted that the clay content of these samples is between 9–11%.

### Fluids

Previous studies have shown that clay swelling and fines migration is one reason for permeability reduction during fluids invasion^35^. To exclude or minimize the influence of clay swelling and fines migration during our experiment, we use synthetic brine with total salinity of 38.0 g/L as injection fluid to match the formation brine. Mineral ions contents of the synthetic brine are listed in Table 2.

**Table 2:** Mineral ion content of synthetic brine.

Mineral ions	Na^+^ + K^+^	Ca^2+^	Mg^2+^	SO_4_^2−^	Cl^−^	HCO_3_^−^
Concentration/(mg·L^−1^)	5984	5821	245	149	19316	318

In addition, we choose fluorinated oil as simulated oil. One reason for using fluorinated oil is that no NMR signals can be detected in fluorinated oil, that is to say, all signals collected by NMR apparatus are from aqueous phase^36^. Under this condition, original oil distribution and aqueous phase migration in rock samples can be determined by comparing T_2_ spectrum differences before and after shut-ins. Another reason for using fluorinated oil is that it doesn’t interact with the rock sample and synthetic brine. Density and viscosity of fluorinated oil is 1.8 g/cm^3^ and 2.1 mPa·s at 25 °C.

### Core flooding procedure

**Figure 2:**
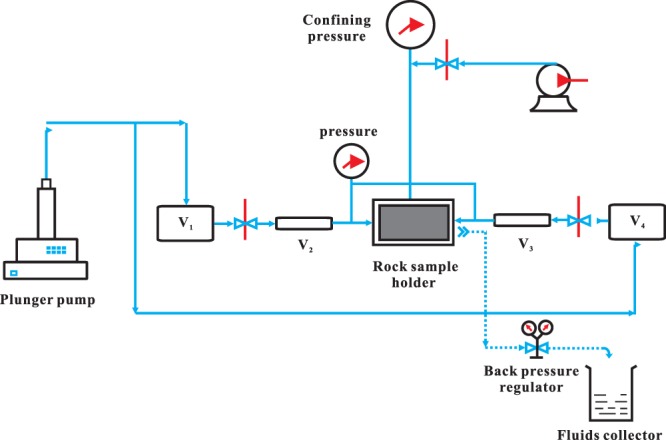
Schematic of the experimental setup. A core-flooding sequence is used to simulate a hydraulic fracturing process including fluid invasion, well shut-ins and flowback.

**Figure 3:**
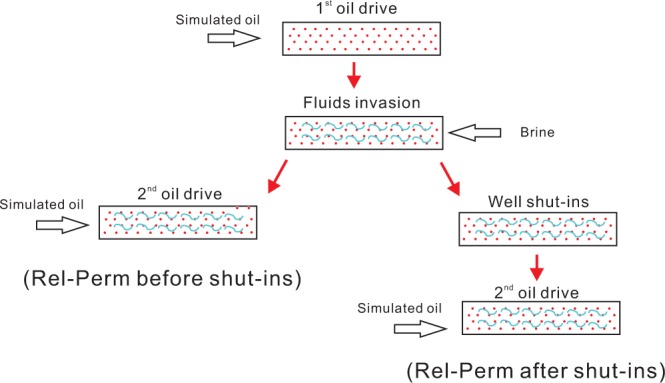
Core flooding procedure. This scheme is to simulate hydraulic fracturing, water invasion, well shut-in and flow-back by injecting different fluids from different ends of rock samples, aiming to obtain relative permeability before and after well shut-ins.

In the field operation of hydraulic fracturing, a portion of fluids would invade into formation matrix under high pump pressure. After that, when well bottom hole pressure is less than reservoir pressure, hydrocarbons flow back into fracture and wellbore. Here in laboratory conditions, we simulate this process by injecting different fluids from different ends of the rock sample. As shown in Figs 2 and 3, the experimental procedure can be divided into 4 steps: 1^st^ oil drive, fluid invasion, well shut-ins and 2^nd^ oil drive.

**Step 1: 1st oil drive**. Mount a dry and evacuated core sample into core holder at 10 MPa confining pressure, detect the sealing capacity of the entire system and vacuum the rock sample using a vacuum pump. When the pressure at vacuum gauge can stay constant, larger than 0.09 MPa and no longer fall down, we inject simulated oil at a constant flow rate of 0.1 mL/min. When rock sample is fully saturated with simulated oil, record fluid flow rate and pressure drop along the core sample. Oil phase absolute permeability of the core is measured during this step.

**Step 2: Fluid invasion**. Inject 1 PV synthetic brine from the right end of the rock sample at 0.1 mL/min to simulate fluids invasion during hydraulic fracturing. Then inject fluorinated oil from the left end at the same rate, calculate the relative permeability for simulated oil before well shut-ins. After that, repeat fluid invasion process.

**Step 3: well shut-ins**. Revoke all pressures to ensure there are no further pressure gradients across rock sample. Rest rock samples for a different length of time to simulate well shut-ins. During this period, capillary dominated spontaneous imbibition began to play a role, phases in rock sample redistribute under counter-current imbibition mechanism.

**Step 4: 2**^**nd**^ **oil drive**. Inject simulated oil from the left end and displace the injected brine in an opposite direction. Same as the above injection, a controlled amount of simulated oil was injected at a constant flow rate of 0.1 mL/min. The injection period will terminate if pressure across the rock sample reaches a plateau and only simulated oil is flowing out. During this process, record and store pressure into the computer, collect the flow-back brine into the graduated cylinder and calculate the relative permeability of simulated oil at the residual water saturation.

### NMR detection principle

Low-field NMR analysis apparatus is used to obtain an attenuation signal of spin echo string via testing Carr-Purcell-Meiboom-Gill (CPMG) pulse sequence^37^. This signal is the superimpose of hydrogen atom (^1^H) coming from the fluids saturated in porous media. The magnitude attenuation of the spin echo string can be precisely fitted with a sum set of exponential decay curves. Each exponential curve has a different attenuation constant, and the set of all the attenuation constants form the transverse relaxation time (T_2_) distribution. When we put a rock sample saturated with oil and/or water into a low magnetic field, energy transition, absorption and release will occur after absorbing a specific frequency of radiofrequency pulse and a strong interaction between ^1^H (in porous media fluids) and magnetic field (produced by NMR instrument) will happen. Low magnetic field NMR method can be used in research of pore water content, pore fluids distribution and pore water migration, it can also be used in the study of aqueous phase migration in porous media during well shut-ins. According to previous studies^38^, NMR transverse relaxation time can be expressed as

1 $${T}_{2}=\frac{1}{{F}_{s}{\rho }_{2}}r$$

where *T*_2_ is transverse relaxation time (ms), *F*_*S*_ is the dimensionless shape factor for a pore, *ρ*_2_ is the surface relaxivity (μm/ms), *r* is pore radius (μm). When a given core is considered, its shape factor (*F*_*s*_) and surface relaxivity (*ρ*_2_) can be assumed to be constant. Thus,

2 $${T}_{2}=C\times r$$

where $C=1/({F}_{S}{\rho }_{2})$(ms/μm), and *C* is a constant conversion coefficient (ms/μm). Hence, if the relationship between pore radius and T_2_ can be found, NMR T_2_ spectrum can be converted to pore radius distribution curve and the inside pore fluids migration can be evaluated by NMR data.

From equation (2) we can see that relaxation time can reflect pore size, larger relaxation time corresponds to larger pore radius. At the same time, magnetic moment produced by ^1^H can be presented by macroscopic magnetization vector, and this vector is in proportional to the number of ^1^H and the amount of porous fluids. Meanwhile, macroscopic magnetization vector can be described as NMR signal amplitude in T_2_ spectrum. Accordingly, the larger the NMR signal amplitude, the larger the amount of fluids in porous media.

### NMR detection procedure

Core sample cq002-1 and cq002-2 will be used in low magnetic field NMR detection, firstly, we inject 10 PV fluorinated oil into the rock sample from the left end to achieve 100% oil saturation. Secondly, we inject 0.2 PV formation brine via the right end. After that, we remove the rock sample from the core holder and wipe the attached water, seal the core sample by wrapping it up using a plastic membrane and put it into a test tubing. This step will reduce water evaporation and increase measurement accuracy during long time testing. Lastly, we put the test tubing and the core sample into NMR apparatus to obtain T_2_ spectrum periodically at different shut-in time. In order to distinguish T_2_ curve differences more clearly, we choose 0 h, 20 h, 40 h, and 90 h as testing time nodes, which is much longer than that in the core-flooding experiment.

In the low field NMR detection, a MacroMR12-150H-VTHP type apparatus (Niumai instrument co., China) and a GPMG pulse sequence method were applied. The magnetic field intensity in our experiment is 0.52T, which is very small and suitable for low permeability core samples. In addition, the waiting time, echo time, echo number and scanning number were set as 3000 ms, 300 μs, 1024 and 64, respectively. To reduce the liquid loss caused by evaporation, the whole experiment is performed at 25 ± 1 °C. Other methods that can increase the signal/noise ratio (SNR) and results accuracy include independent radiofrequency emission and reception systems.

## Results and Discussion

### SEM results before and after soaking in formation brine

**Figure 4:**
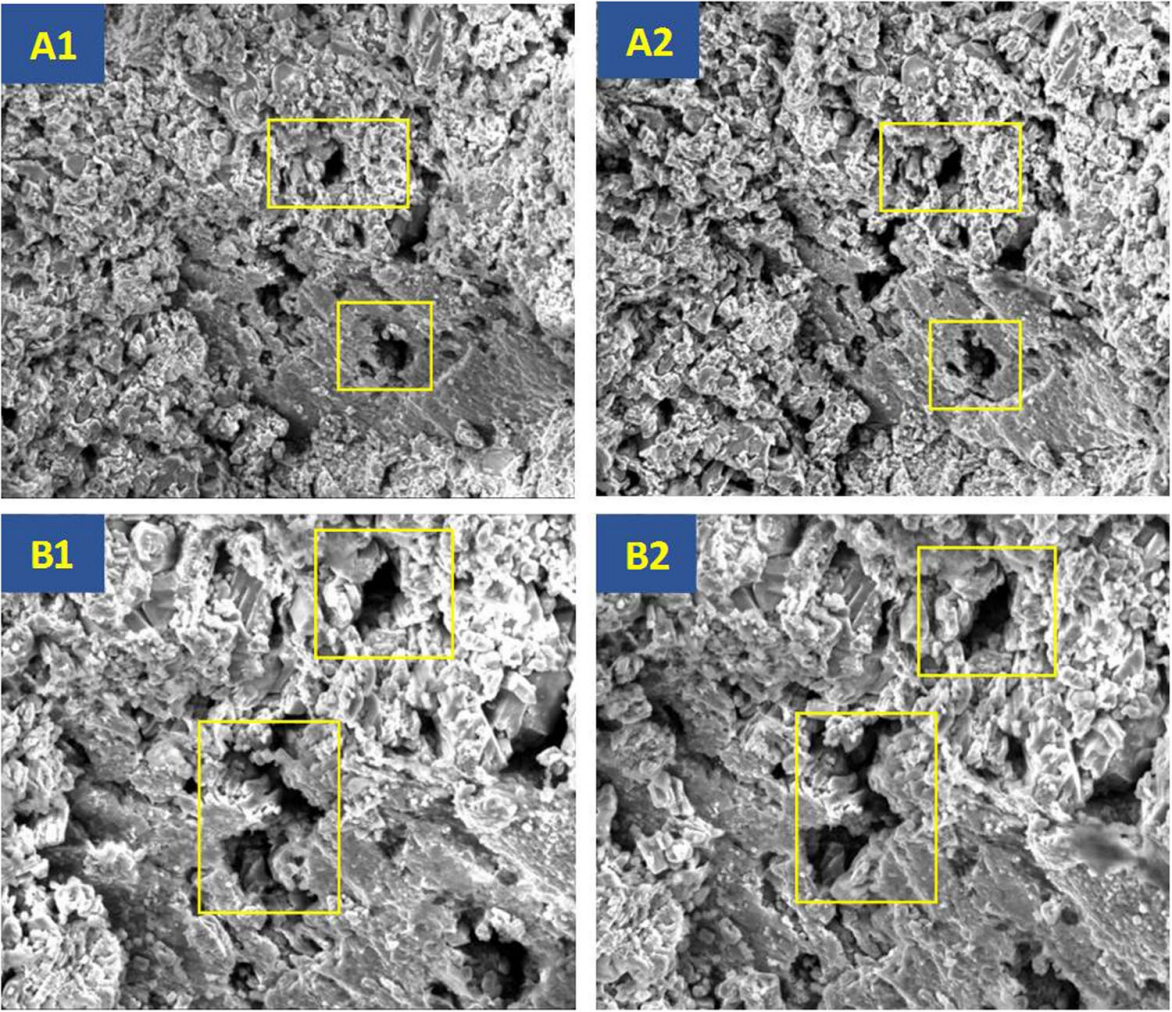
SEM images before and after soaking in formation brine. SEM results showed that no significant clay swelling and fines migration happened when rock sample was soaked in synthetic brine.

As illustrated in the above section, we need to exclude the influence of clay swelling and fines migration in our experiment. To examine the influence of synthetic brine on the rock samples, we use the SEM detection method. We scan the same positions on a rock slice in 600 magnification before and after it was soaked in brine for 12 h. Figure 4 shows SEM results for two different positions (position A and position B), figures A1 and B1 are images before soaking while A2 and B2 are images after soaking. Through the comparison of A1 and A2, B1 and B2, we can find that clay swelling and fines migration do happen after soaking in formation brine, while only in a very slight degree and this tiny level of fines migration has not blocked the original pore spaces. Under this condition, it is safe to say that the synthetic brine we used can help to exclude the influence of clay swelling in our following experiment.

### Relative permeability enhancement after shut-ins

**Figure 5:**
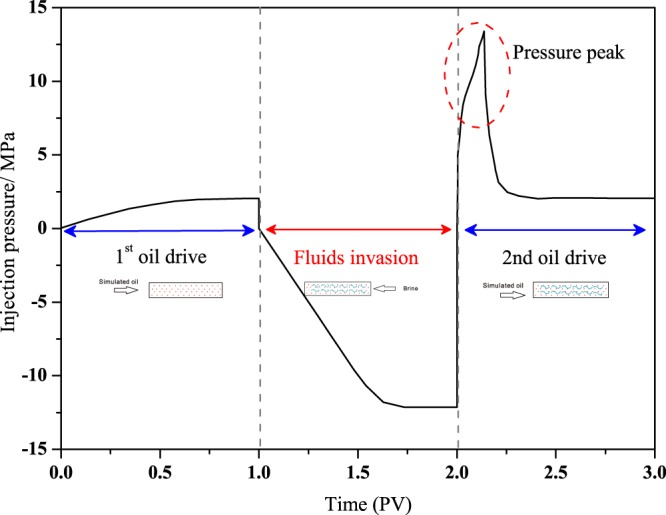
Pressure across the core sample during fluids injection. In 1^st^ oil drive (step 1), pressure rise linearly at the beginning then reaches a plateau of 1.97 MPa. In fluids invasion (step 2), pressure also increases with time while having a greater slope before reaching a plateau. In 2^nd^ oil drive (step 4), pressure curve vibrates and presents a drastic pressure peak and a pressure drop area.

Figure 5 shows an example of a typical pressure curve across the rock sample in the whole experiment procedure. At the beginning of 1^st^ oil injection (**Step 1**), pressure rises linearly with time, indicating this is a piston displacement^39^. After about 0.69 PV simulated oil injection, pressure gradually reaches a plateau of 1.97 MPa. When pressure difference becomes smaller and the pressure plateau become more stable, at the end of 1 PV injection, we stop 1^st^ oil drive process.

In step 2, we inject synthetic brine in a reverse direction to simulate fluids invasion, we draw its pressure curve in negative coordinates and we can see that the pressure curve also increases with time before 0.54 PV brine injection, and then gradually get to a plateau. Comparing to Step 1, pressure curve of step 2 has a greater slope.

**Step 3** is well shut-ins period (this process was not shown in Fig. 5), well shut-ins period last 6 h, 12 h, 18 h and 24 h for the four tested core samples. During this step, we remove all displacing and confining pressure to ensure there is only capillary dominated spontaneous imbibition. When it turns to 2^nd^ oil drive (**Step 4**), we injected simulated oil from left end again to simulate the flow-back process. In this section, the pressure curve increases fast and vibrates sharply at the beginning and presenting a pressure peak area in each case, after that, pressure curve dropped fast to a plateau. When there is no water production detected at the right end, we use that pressure plateau to calculate the relative permeability via Darcy’s law.

**Figure 6:**
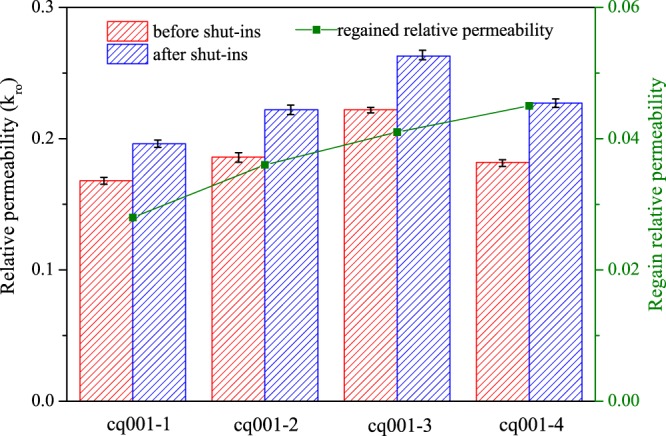
Relative permeability of simulated oil before and after well shut-ins for rock samples. **c**q001-1: 6 h shut-in time; cq001-2: 12 h shut-in time; cq001-3: 18 h shut-in time; cq001-4: 24 h shut-in time. Well shut-ins can increase rock sample permeability and this improvement depends on the length of shut-ins duration.

To quantify permeability enhancement after well shut-ins, we calculate the relative permeability before and after well shut-ins and the results are shown in Fig. 6. The average water saturation is 0.58 after brine invasion while is 0.47 after 2^nd^ hydrocarbon flood, and this average water saturation is where we calculate relative permeability to simulated oil. In Fig. 6, red column shows relative permeability of simulated oil before well shut-ins, blue column shows permeability after well shut-ins. Note that before well shut-ins, rock samples have relative permeability ranging from 0.16 to 0.23 after brine invasion, while after different length of well shut-ins, permeability shows varying degrees of enhancement from 0.19 to 0.27. In olive dot-line, we draw the regained relative permeability and we can see that permeability increase fast at the beginning of shut-ins while this increasing rate gradually slows down during long time shut-ins. At a low fluids flow rate of 0.1 mL/min, rock sample permeability reduces in a relatively large degree after water invasion, while it can recover to some extent after shut-ins. Well shut-ins can increase rock sample permeability and this improvement depends on the length of shut-ins duration.

### T_2_ spectrum migration during shut-in period

After the comparison of core sample mercury injection curve and NMR T_2_ spectrum, related authors gave an improved method to transfer T_2_ spectrum distribution to pore radius distribution and calculated the transfer parameter *C* in equation (2) ^40,41^. According to their previous work, we estimate the relationship between T_2_ relaxation time, pore radius and pore type as in Table 3 and we will use this relationship in the latter discussion.

**Table 3:** Relationship between T_2_ relaxation time and pore characteristics.

T_2_ relaxation time/ms	Pore radius/μm	Pore type
T_2_ relaxation time ≤ 1	pore radius ≤ 2	micropore
1 < T_2_ relaxation time ≤ 10	2 < pore radius ≤ 10	small mesopore
10 < T_2_ relaxation time ≤ 50	10 < pore radius ≤ 20	mesopore
50 < T_2_ relaxation time	20 < pore radius	macropore

**Figure 7:**
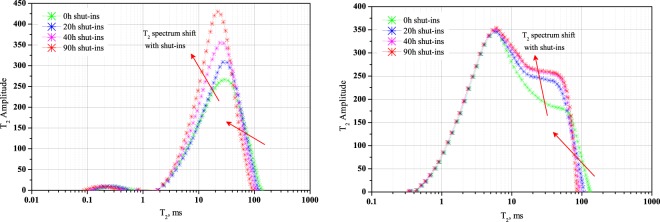
T_2_ spectrum shift during shut-ins period, drawn in a semi-logarithmic coordinate system. (**a**) Core sample cq002-1; (**b**) core sample cq002-2. After different length of shut-ins periods, (1) T_2_ spectrum curves shift towards left side, (2) T_2_ amplitude at small intervals increasing.

Figure 7 shows the T_2_ spectrum distribution of core sample cq002-1 and core sample cq002-2 at different shut-in time after brine injection, green line shows T_2_ spectrum at the end of fluids injection, we set this time node as 0 h shut-ins. The other blue, magenta and red lines show T_2_ spectrum after 20 h, 40 h and 90 h’s shut-ins. In Fig. 7a, T_2_ spectrum shows two amplitude peaks, a small one in the intervals of 0.1-1 ms and a large one in the intervals of 1–100 ms. Note that these two peaks are not well connected indicating the disconnection of micropores and mesopores. Whereas in Fig. 7b, T_2_ spectrum only have one long-span amplitude peaks, in the intervals of 0.1 ms to 100 ms. In these two figures, the same curve shift tendency can be seen as red arrow indicates. After different length of shut-ins, one phenomenon is T_2_ spectrum shift towards left side, the other one is T_2_ amplitude in small pore radius increases. For example, in Fig. 7a, after 90 h’s shut-ins, the right end of T_2_ spectrum shift from 138.1 ms to 94.2 ms and T_2_ amplitude at 22.1 ms increases from 258.9 to 430.2.

As illustrated above, pore radius and T_2_ relaxation time are in a proportional relationship, a larger pore radius has longer T_2_ relaxation time. Moreover, fluids content in pore spaces corresponds to T_2_ amplitude; higher T_2_ amplitude means higher fluids content. Under these circumstances, we can see that most of the injected brine distribute in macropores and mesopores at the beginning of water invasion. During shut-ins periods, synthetic brine which originally existed in intervals of 90–140 ms moves to intervals of 10–50 ms, migrating from large pore spaces to relative smaller ones. Aqueous migration results in the increase of water content in smaller pore spaces and this is the reason why T_2_ amplitude at smaller intervals increases.

**Figure 8:**
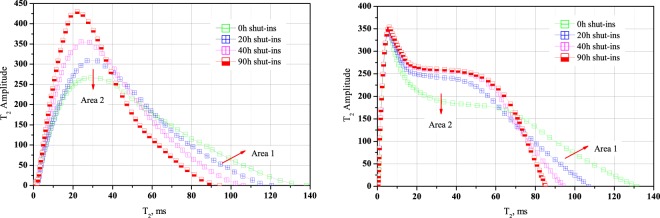
T_2_ spectrum shift during shut-ins period, drawn in an ordinary coordinate system. (**a**) Core sample cq002-1; (**b**) core sample cq002-2. The main trend of aqueous phase migration is from macropores/mesopores to small mesopores/micropores.

To intuitively understand fluids distribution in macropores and mesopores, as shown in Fig. 8, we draw T_2_ spectrum map in an ordinary coordinate system for core sample cq002-1 and core sample cq002-2. It shows that T_2_ amplitude peak appears at intervals of 10–20 ms while most of the integral area distribute at intervals of 20–60 ms, mainly in mesopores and macropores. Pore fluids migration can also be investigated via the shift of T_2_ spectrum in this figure, for example, in Fig. 8a, after 20 h shut-ins, T_2_ spectrum migrates from green line to blue line, fluids originally distributed in *Area 1* now migrates to *Area 2*. The same situation can also be applied to core sample cq002-2, the main aqueous migration trend is from macropores and mesopores to small mesopores and micropores.

**Figure 9:**
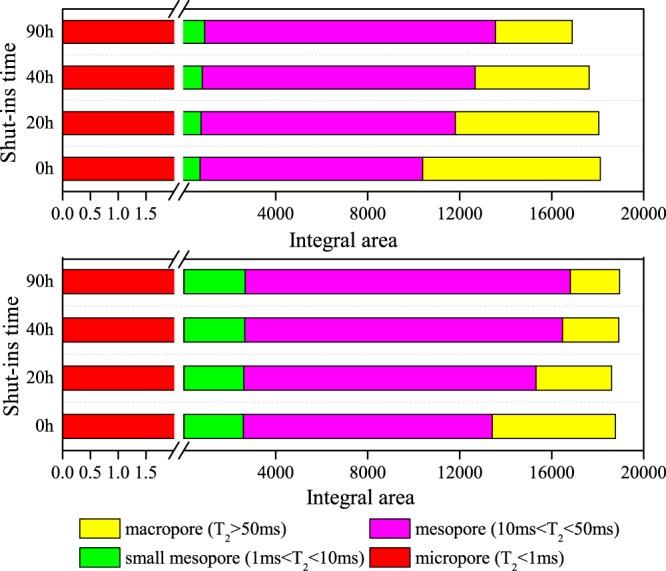
Integral areas under T_2_ relaxation time. (**a**) Core sample cq002-1; (**b**) core sample cq002-2. (1) Most of the aqueous phase distribute in macropores and mesopores after water invasion. (2) During well shut-ins, integral area in macropores decreases while in mesopores increase. (3) The main aqueous phase migration direction in shut-ins duration is from macropores to mesopores.

To quantitative characterize aqueous phase migration during shut-ins period, we calculated the integral area under T_2_ relaxation time for different shut-ins. As discussed above, T_2_ transverse relaxation time can be representative for pore radius, T_2_ amplitude can be representative for aqueous content in pore radius, and then the integral area under T_2_ transverse relaxation time can be representative for fluids volume in pore spaces. As shown in Fig. 9, we draw the integral area of micropores, small mesopores, mesopores and macropores in different colors, we can see that integral area of macropores and mesopores is the largest, taking up a proportion more than 95%, while integral area of small mesopores and micropores only take up less than 5%. This indicates that most of the injected aqueous phase distribute in mesopores and macropores after fluids invasion. Taking core sample cq002-1 as an example (Fig. 9a), at the beginning of well shut-ins, integral areas at micropores and small mesopores are 2.85 and 716.85, while integral areas at mesopores and macropores are 9676.08 and 7712.69. Then after 20 h, 40 h and 90 h’s well shut-ins, integral area at macropores decreased to 6241.59, 4950.0 and 3332.31, while area at mesopores increased to 11052.60, 11860.12 and 12640.10. It should be noted that the integral area at micropores and small mesopores also increased while it did not show an obvious increment. The main aqueous phase migration direction during shut-ins period is from macropores to mesopores.

### Why shut-ins can increase permeability

Previous literatures have revealed that fluids loss and water invasion during hydraulic fracturing can cause serious permeability damage near fracture faces. Aqueous phase invasion into formation can cause clay swelling, solid precipitation, and fines migration and result in permeability reduction physically. In addition, the invading aqueous phase can form a water-block area near fracture face, increasing capillary discontinuity and blocking hydrocarbon phases flow channel. What’s more, aqueous phase and previous existing hydrocarbon phase can form a 2-phase seepage resistance, which will drastically decrease effective permeability for hydrocarbon phase. In our experiment, we tried to exclude the influence of clay swelling and fines migration by using brine injection, in these cases, we focus on aqueous phase migration, near fracture water-block removal and capillary issues.

**Figure 10:**
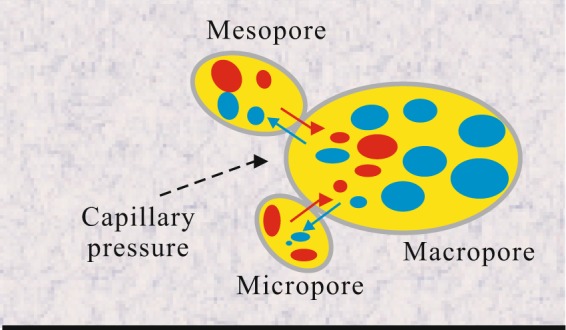
Aqueous phase redistribution and migration during shut-ins. Aqueous phases redistribute and migrate to smaller pore spaces and inner distances, remove water-blocks, reduce capillary discontinuity and form channels for oil phase flow.

Our experiment has shown that permeability enhancement happens after well shut-ins and this is mainly due to aqueous phase redistribution and migration during shut-ins. Aqueous phase redistribute and migrate to smaller pore spaces and deeper distances can benefit to enhance matrix permeability and the subsequent hydrocarbon outputs because (1) it removes near fracture water-block. After hydraulic fracturing, a large amount of water is trapped near the fracture face and these trapped water mainly distribute in macropores and mesopores. Trapped aqueous phase in macropores and mesopores will form a water-block area, increasing seepage resistant for hydrocarbon phase. For depletion developed well, drawdown pressure has to be large enough to overcome these water-blocks to drain oil/gas from the matrix to hydraulic fractures. As shown in a cartoon map of Fig. 10, during shut-ins period, aqueous phase migrates from larger pore spaces to smaller ones, displacing hydrocarbon phase via capillary dominated counter-current imbibition. Aqueous phase redistribution and migration can free up large pore spaces, weaken or even remove water-block. (2) It reduces capillary discontinuity. Trapped water mainly distributes around fractures as a large contiguous aqueous phase and block channels for oil/gas flow. Well shut-ins and water redistribution lead these aqueous phases no longer distributed as a contiguous whole, some of them went into smaller pore spaces, some went to deeper distances, and thus reduce seepage resistance for hydrocarbon phase. (3) It increases the relative permeability of hydrocarbon phase. Aqueous phase imbibed into the matrix and displace oil/gas out in an opposite direction, and this is counter-current imbibition. Imbibition mechanism can increase the amount of hydrocarbon phase and decrease the amount of aqueous phase in pore spaces, and simultaneously increase the relative permeability of hydrocarbon phase by changing water saturation.

## Conclusion

In this paper, we investigated permeability reduction after water invasion and permeability enhancement after well shut-ins using a core flooding system. In addition, we studied aqueous phase migration in shut-ins period using NMR method intending to reveal the inner mechanism. Core flooding experiments showed that fluid loss and aqueous phase invasion into formation can cause matrix permeability reduction, while well shut-ins can regain permeability. Well shut-ins increase rock effective permeability and this improvement depends on the length of shut-ins. NMR results showed that aqueous phase mainly distributes in macropores and mesopores after water invasion, while during shut-ins period, these aqueous phases redistribute and migrate from larger pore spaces to smaller ones and their main migrate direction is from macropores to mesopores. Aqueous phase redistribution and migration during shut-ins period can remove water-block, reduce capillary discontinuity and increase hydrocarbon phase relative permeability, and this is one reason for permeability enhancement and hydrocarbon output improvement after well shut-ins.
